# Novel X-ray image noise reduction technology reduces patient radiation dose while maintaining image quality in coronary angiography

**DOI:** 10.1007/s12471-015-0742-1

**Published:** 2015-09-14

**Authors:** T. ten Cate, M. van Wely, H. Gehlmann, M. Mauti, C. Camaro, N. Reifart, H. Suryapranata, M.J. de Boer

**Affiliations:** 1 Department of Interventional Cardiology, RadboudUMC, Geert Grooteplein-Zuid 22, 6525 Nijmegen, GA The Netherlands; 2 Philips Healthcare, Best, The Netherlands; 3 Main-Taunus-Privatklinik, Bad Soden, Germany

**Keywords:** Interventional cardiology, Safety, Image processing, DAP, Dose reduction

## Abstract

**Aims:**

The consequences of high radiation dose for patient and staff demand constant improvements in X-ray dose reduction technology. This study assessed non-inferiority of image quality and quantified patient dose reduction in interventional cardiology for an anatomy-specific optimised cine acquisition chain combined with advanced real-time image noise reduction algorithms referred to as ‘study cine’, compared with conventional angiography.

**Methods:**

Fifty patients underwent two coronary angiographic acquisitions: one with advanced image processing and optimised exposure system settings to enable dose reduction (study cine) and one with standard image processing and exposure settings (reference cine). The image sets of 39 patients (18 females, 21 males) were rated by six experienced independent reviewers, blinded to the patient and image characteristics. The image pairs were randomly presented. Overall 85 % of the study cine images were rated as better or equal quality compared with the reference cine (95 % CI 0.81–0.90). The median dose area product per frame decreased from 55 to 26 mGy.cm^2^/frame (53 % reduction, *p* < 0.001).

**Conclusion:**

This study demonstrates that the novel X-ray imaging technology provides non-inferior image quality compared with conventional angiographic systems for interventional cardiology with a 53 % patient dose reduction.

## Introduction

Prolonged X-ray guided procedures are associated with a risk of deterministic and stochastic injury [1, 2]. Although the number of percutaneous coronary interventions (PCI) has remained relatively stable in recent years, the complexity of coronary interventions has increased. One contributor to prolonged procedures is PCI of chronic total occlusions, which has become increasingly successful [3–5]. Prolonged procedures result in higher radiation doses [6–8], especially in obese patients, in whom higher radiation is often necessary to obtain adequate diagnostic images [9, 10].

In accordance with the ALARA (As Low As Reasonable Achievable) principle, the best ratio between image quality and radiation dose should be determined. Dose awareness and recent developments in noise reduction algorithms have created new opportunities for dose reduction without compromising image quality [11–13].

Recently, a novel X-ray imaging technology that combines advanced real-time image noise reduction algorithms with state-of-the-art hardware to significantly reduce patient radiation dose for fluoroscopy and cine acquisition in interventional cardiology became available (AlluraClarity; Philips Healthcare, Best, the Netherlands). The complete acquisition chain has been optimised (e.g. grid switch, beam filtering, pulse width, spot size, detector and image processing engine, etc.). Moreover, smaller focal spot and shorter X-ray pulses are used to further enhance image quality.

The novel X-ray imaging technology showed a 40 % procedural dose reduction for patients undergoing complex ablations and 50 % dose reduction to the main operator [14] in electrophysiology.

In interventional neuroradiology, non-inferior image quality was shown for digital subtraction angiography (DSA) with a 75 % patient radiation dose reduction [12] and 60 % total procedural dose reduction with similar procedural characteristics[13].

The current study was designed to assess whether this novel X-ray imaging technology that reduces patient dose allows for cine image acquisition without loss of diagnostic image quality in coronary angiography.

## Methods

The study was conducted in accordance with the provisions of the Declaration of Helsinki as amended in Edinburgh, Scotland (2008). Each patient signed informed consent. The enrolment of patients occurred between 11 September and 23 November 2012. The study was approved by the local ethics committee and published on clinicaltrials.gov (NCT01684826).

A flat-detector angiography system (Allura Xper FD10; Philips Healthcare, Best, the Netherlands) equipped with standard image processing and exposure system settings (100 % dose) was used. For the purpose of this study, this X-ray system was also equipped with the advanced image processing and optimised exposure system settings to enable patient dose reduction (ClarityIQ; Philips Healthcare, Best, the Netherlands). The study was designed to assess non-inferiority of image quality and to quantify patient dose reduction between a cine acquisition run acquired with the standard image processing and exposure settings (reference cine) compared with a cine acquisition run acquired with the advanced image processing and optimised exposure system settings to enable dose reduction (study cine).

### Improvements in image processing and acquisition chain

The X-ray acquisition chain for the study cine was modified to enable patient radiation dose reduction. Increase in copper filtration for the study cine was implemented (0.1 mm Cu and 1 mm Al) compared with the reference cine (0.0 mm Cu and 0.0 mm Al). No changes were applied in kV-mA-ms controls and frame speed, while the detector dose was reduced.

In interventional cardiology, image processing is more challenging when compared with DSA due to the movement of the heart. The advanced real-time image processing combines several features that enhance image quality [12–14]. The spatial noise reduction algorithm uses the random nature of noise to distinguish between the clinical information and the noise in a single image. The algorithm filters out the noise by averaging the pixel intensity with the surrounding pixels. For temporal noise reduction it is essential to detect motion between frames to avoid the appearance of ghost images of moving objects such as catheters. The novel technology uses motion compensation to align moving objects before averaging. This allows for more consecutive images to be averaged and thus reduces noise significantly.

### Patients

Patients older than 18 who were referred for elective invasive diagnostic coronary angiography were enrolled. Exclusion criteria were known kidney dysfunction (eGFR < 60 ml/kg/min), participation in other clinical trials, known pregnancy and breastfeeding. In order to depict a realistic sample of the population, body mass index (BMI) was not used as a selection criterion. Therefore, also obese patients could be included in the study.

### Image acquisition

Four interventional cardiologists performed the procedures. For the evaluation of image quality, the left coronary artery was used. The acquisition was done in a left anterior oblique (LAO) and cranial projection angle, an LAO of 45 degrees and a cranial angle of 20 degrees were advised. However, when the coronary artery could not be properly viewed the operator was free to change the angulations accordingly.

Two subsequent runs were acquired, the first with the reference settings followed by a run with the study settings. Contrast dye injection was performed with hand injection using a 10 ml syringe and a 5 French diagnostic catheter. The images were acquired during breath hold with the table in a stable position during acquisition. Table height, tube angulation, field of view and gantry settings were left the same for both image acquisitions.

### Patient radiation dose evaluation

For each run the number of frames per run, cumulative dose area product (DAP) and Air Kerma (AK) values as indicated by the X-ray system were entered in the case report form. Average DAP and AK per frame were calculated after the procedure to correct for variation in run duration between the two acquisitions.

### Image analysis

All images were stored offline and evaluated by six independent reviewers from five hospitals in Europe. All have at least 10 years of experience with acquiring and reviewing coronary angiograms and were not involved in the acquisition of the two study runs.

For the review, the two acquisitions per patient were displayed in pairs, side by side, on two diagnostic quality image review monitors (A and B, Philips MML1942-PER). The images were presented to the reviewers in a random order. The reviewers were blinded to patient and image characteristics but post-processing adjustments were allowed.

Image quality was assessed based on general appearance, ability to assess large arteries, side branches and their origin, ability to assess visual stenosis and other clinically relevant information. Also, the reviewers were asked to assess the image quality taking into account noise levels, surrounding tissue (such as lung or liver) and image artifacts.

### Statistical analysis

All analysis were conducted using SAS/STAT® software. The primary objective of this study was to demonstrate non-inferior clinical image quality of the study settings compared with the reference settings, with the null hypothesis: Ho: ρ ≤ 0.80 and alternative hypothesis: Ha: ρ > 0.80, with ρ being the proportion of images rated as having equal or better clinical quality for study cine images compared with the reference cine images.

Radiation dose was analysed as averaged dose per frame. Descriptive statistics for dose per frame are presented for the study and reference cine acquisitions. Differences in radiation dose between the two acquisitions was evaluated with the paired two-sided Student t-test and the non-parametric Wilcoxon signed-rank test. *P* < 0.05 was considered statistically significant.

## Results

### Patients

A total of 50 patients were enrolled in the study. Eleven (11) were left out of the analysis: one patient withdrew informed consent, in 2 patients the images required for the study were not acquired due to medical reasons, 2 patients missed the dose data for a run and in 6 patients the images could not be retrieved for off-line comparison. Evaluable data for image quality assessment and patient radiation dose analysis were available for 39 patients. Patient characteristics and procedure indications are listed in Table 1; 10 patients had a BMI > 28 kg/m^2^. Most patients (71.8 %) had a positive family history for premature coronary artery disease, 59 % had hypertension.

**Table 1:** Patient and procedure characteristics

		Rated (*n* = 39)
Age (years)	Mean (SD)	63.4 (10.7)
Gender	Female (*n*, %)	18 (46.2 %)
	Male (*n*, %)	21 (53.8 %)
BMI (kg/m^2^)	Mean (SD)	26.4 (3.3)
	Median	26.1
Procedure indication	Diagnostic	30 (76.9 %)
	Intervention	9 (23.1 %)
Vessels treated	Left main stem	1 (2.6 %)
	LAD	5 (12.8 %)
	LCX	4 (10.3 %)

*SD* standard deviation, *BMI* body mass index.

### Image quality

**Fig. 1:**
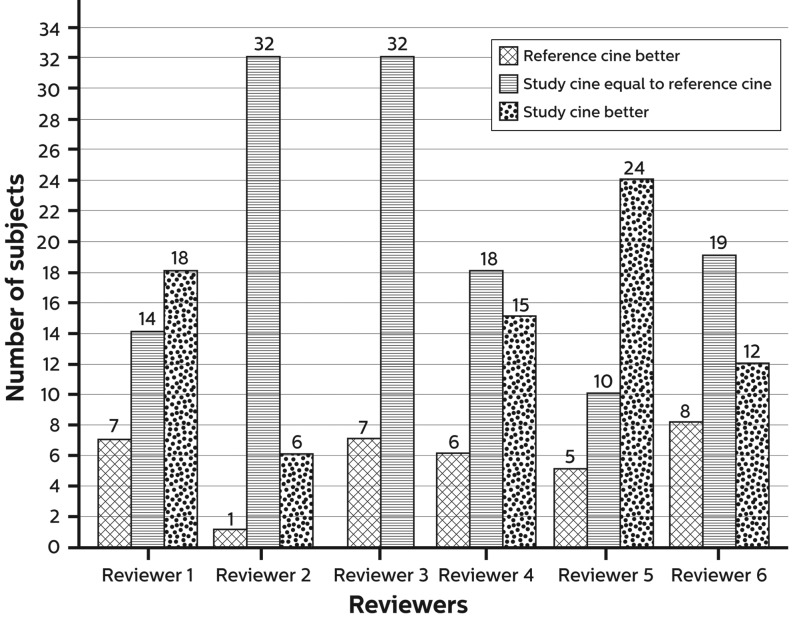
Summary of image assessment

**Fig. 2:**
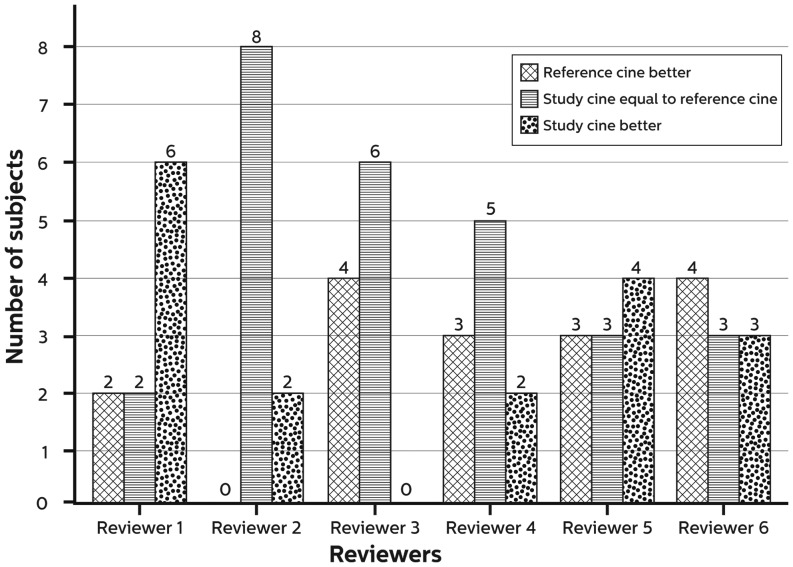
Summary of image assessment of patients with a BMI > 28 kg/m^2^

**Fig. 3:**
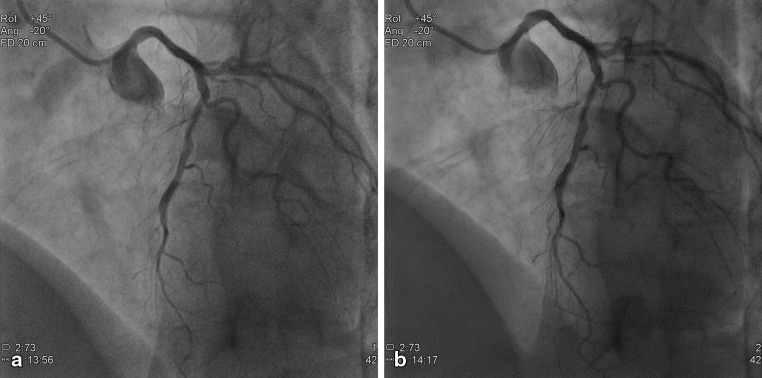
Example of a 55-year-old female (BMI 30.2 kg/m^2^), current smoker with a medical history of hypertension, hyperlipidaemia and a family history of premature coronary artery disease (CAD). The patient already underwent PCI (stent in LAD and RCA) and was admitted for diagnostic procedure for complaints of progressive angina without symptoms at rest. The top panel, **a** represents the reference image and **b** the study image. Cine acquisitions obtained at 15 fps, field of view 20 cm, LAO 45 CRA 20 with (**a**) reference cine (*DAP*: 60.40 mGy.cm^2^/frame), (**b**) study cine (*DAP*: 27.01 mGy.cm^2^/frame)

All images acquired with the study cine were of diagnostic quality to assess clinically relevant information. Overall, 85 % of the image sets were considered of better or equal (non-inferior) image quality compared with the reference cine (95 % CI 0.81–0.90) with the study cine considered better in 32 % of the images. Figs. 1 and 2 show the rating of the images in more detail for the complete group and for patients with BMI > 28 kg/m^2^, respectively. Fig. 3 shows an example of a study patient.

### Radiation exposure

Table 2 shows the radiation dose characteristics of the 39 analysed patients. Data are presented as dose averages/frame of the cine acquisitions. The average number of frames per run was 73.2 ± 28 for the study cine vs 73.9 ± 32.4 for the reference cine. Overall, the median reduction was 53 % for both DAP and AK (*p* < 0.001). The patient dose reduction for obese patients (BMI > 28 kg/m^2^) is presented separately in Table 3.

**Table 2:** Radiation characteristics for the complete study population

		Study cine	Reference cine	*p* value
AirKerma [mGy/frame]	*N*	39	39	
	Mean (SD)	0.44 (0.1)	0.95 (0.2)	< 0.001
	Median	0.44	0.94	< 0.001
DAP [mGy.cm^2^/frame]	*N*	39	39	
	Mean (SD)	26 (5.7)	55 (6.9)	< 0.001
	Median	26	55	< 0.001

*SD* standard deviation, *DAP* dose area product.

**Table 3:** Radiation characteristics for patients with BMI > 28 kg/m2

		Study cine	Reference cine	*p* value
AirKerma [mGy/frame]	*N*	10	10	
	Mean (SD)	0.5 (0.1)	0.95 (0.1)	< 0.0001
	Median	0.48	0.97	
DAP [mGy.cm^2^/frame]	*N*	10	10	
	Mean (SD)	31 (5.7)	57 (10.4)	< 0.0001
	Median	31	59	

*SD* standard deviation, *DAP* dose area product.

## Discussion

The results of this study demonstrate that the novel X-ray image acquisition technology allows for 53 % radiation reduction for cine acquisition without compromising image quality.

A wide variation in radiation dose delivered to patient and staff for the same type of fluoroscopically guided procedures has been reported [15, 16]. This seems mostly attributable to differences in equipment performance, way of working, complexity of the procedure and patient size.

Coronary angiography and PCI procedures are among the procedures that account for the highest radiation dose to patients. In the SENTINEL survey a total DAP of 45 Gy.cm^2^ for coronary angiography and 85 Gy.cm^2^ for PCI was reported [17], in the NEXT survey this was 85 and 193 Gy.cm^2^ respectively [6]. Total DAP is the DAP for fluoroscopy and cine combined. Although fluoroscopy is extensively used during procedures, the total contribution to the total procedure dose is only 25 % for coronary angiography and 50 % for PCI, with an overall contribution of 40 % [18, 19]. Therefore it is important to focus on reduction of the cine dose.

This technology reduces the dose in both fluoroscopy and cine acquisition. However, image quality was assessed during cine acquisition because of the high reproducibility of the two runs and the high contribution of cine to the overall per procedure radiation dose.

The findings of this study are important for the interventional cardiologist, because it can be expected that the observed dose reduction in this study will result in a total procedural dose reduction. This will reduce the risk of stochastic effects as well as skin effects for the patient, and as the patient is the main source of scatter, also will reduce the occupational dose. This is of utmost importance because concerns of radiation exposure for the staff were raised recently [20–23]. Whether dose reduction may also lead to improvements in radiation protection (e.g. lead apron weight) for staff needs further study. Furthermore, patient dose reduction may enable new and complex procedures that currently cannot be performed because the safety limits for radiation exposure are quickly reached.

## Limitations of this study

The main limitation of this study is that comparison was restricted to a single angulation (LAO with cranial angulation). Although the LAO view is commonly used, it has relatively little attenuation from the diaphragm. Further studies are needed to assess the image quality for different tube angulations. Although cine angiography is the largest contributor to total procedural dose, further studies are needed to assess the overall radiation reduction for a procedure using this novel technology. It is expected that reduction in patient entrance dose leads to reduction in operator dose. However, this was not quantified in this study. In addition, image quality assessment was based on a subjective evaluation rather than on objective parameters. This type of side-by-side comparison was previously described and accepted as a good method for subjective but semi-quantitative evaluation of image quality [24].

## Conclusion

The novel X-ray imaging technology provides non-inferior image quality with a 53 % patient dose reduction compared with conventional angiographic systems. This will result in improved patient and staff safety.
